# Chronotoxicity of Acrylamide in Mice Fed a High-Fat Diet: The Involvement of Liver CYP2E1 Upregulation and Gut Leakage

**DOI:** 10.3390/molecules28135132

**Published:** 2023-06-30

**Authors:** Luanfeng Wang, Yanhong Liu, Huajing Gao, Shuqi Ge, Xinru Yao, Chang Liu, Xintong Tan

**Affiliations:** 1 Collaborative Innovation Center for Modern Grain Circulation and Safety, College of Food Science and Engineering, Nanjing University of Finance and Economics, Nanjing 210023, China; wanglf1123@163.com; 2 Key Laboratory of Food Processing Technology and Quality Control of Shandong Higher Education Institutes, College of Food Science and Engineering, Shandong Agricultural University, Taian 271018, China; liuyh20021004@163.com (Y.L.); myszghj@163.com (H.G.); gsqshandong@163.com (S.G.); yumiko020207@163.com (X.Y.); liuchang210403@163.com (C.L.)

**Keywords:** acrylamide, high-fat diet, chronotoxicity, CYP2E1

## Abstract

Acrylamide (ACR) is produced under high-temperature cooking of carbohydrate-rich foods via the Maillard reaction. It has been reported that ACR has hepatic toxicity and can induce liver circadian disorder. A high fat diet (HFD) could dysregulate liver detoxification. The current study showed that administration of ACR (100 mg/kg) reduced the survival rate in HFD-fed mice, which was more pronounced when treated during the night phase than during the day phase. Furthermore, ACR (25 mg/kg) treatment could cause chronotoxicity in mice fed a high-fat diet, manifested as more severe mitochondrial damage of liver during the night phase than during the day phase. Interestingly, HFD induced a higher CYP2E1 expressions for those treated during the night phase, leading to more severe DNA damage. Meanwhile, the expression of gut tight junction proteins also significantly decreases at night phase, leading to the leakage of LPSs and exacerbating the inflammatory response at night phase. These results indicated that a HFD could induce the chronotoxicity of ACR in mice liver, which may be associated with increases in CYP2E1 expression in the liver and gut leak during the night phase.

## 1. Introduction

Acrylamide (ACR) is a known contaminant that can be generated in starchy foods during high-temperature cooking via the Maillard reaction, and is found in some Western-diet foods, such as potato chips and coffee [1,2,3,4]. ACR is toxic to multiple organs and tissues, including the liver, kidney, central nervous system, and reproductive system. It can also lead to skeletal muscle weakness and weight loss in both experimental animal models and humans with long-term exposure [5,6,7]. ACR is mainly metabolized via two pathways in the liver: (a) the glutathione (GSH) pathway, where ACR is catalyzed by glutathione transferase and reacts with GSH to form conjugates, and (b) the cytochrome P450 family 2 subfamily E member 1 (CYP2E1) enzyme pathway. In the latter pathway, ACR can be metabolized to glycidamide (GLY) by CYP2E1, which increases the toxicity of ACR, as GLY consumes 1.5 times more GSH than acrylamide, and GLY also displays genotoxicity by attacking DNA [8,9]. Additionally, a previous study has revealed that ACR induces liver toxicity by inhibiting liver mitochondrial complex I-IV activity [10]. Another study demonstrated that ACR induced a decrease in the mitochondrial membrane potential In human hepatocellular carcinoma HepG2 cells [1].

The circadian rhythm is an autonomous oscillator that regulates many physiological and metabolic processes [11,12]. Liver xenobiotic metabolic enzymes, including cytochrome P450, are also under circadian control [13]. For instance, acetaminophen, a common analgesic, is a chronotoxic substance due to fluctuations in the enzyme that metabolizes it, CYP2E1 [14]. One of our previous studies demonstrated that ACR induced circadian clock alteration, expression of the metabolic enzyme CYP2E1, and P450 oxidoreductase (POR) disorder in the livers of mice [1]. However, it is still unclear whether circadian rhythm alterations could influence ACR chronotoxicity.

Obesity, caused by an energy imbalance between caloric intake and consumption, has become a thorny international health problem worldwide, especially in some Western developed countries [15,16]. A high-fat diet (HFD) has been identified as one of the main causes of obesity. Previous studies have demonstrated that a HFD induces circadian disorder and suppresses the expression of circadian protein Bmal1 in the mouse liver [17,18]. It has also been demonstrated that a HFD increases ACR-induced reproductive toxicity, evidenced by reduced fertility in mice [19]. Moreover, it has been found that HFD-induced obese mice exhibited higher liver CYP2E1 activity that further influenced the disposition kinetics of the muscle relaxant chlorzoxazone in Zucker rats [20]. Of note, a study has revealed that CYP2E1 played a critical role in HFD-induced non-alcoholic steatohepatitis [21]. However, it is unknown whether a HFD affects the hepatotoxicity of toxins such as ACR and other drugs by reprogramming the circadian expression of CYP2E1.

In addition, the Western diet increases the production of lipopolysaccharides (LPSs) by Gram-negative bacteria, which destroys the gut barrier integrity and increases the vulnerability of the intestinal tract to LPS. When excess LPSs enter the circulation they promote systemic inflammation and ultimately compromise liver function [22,23]. Interestingly, the gut tight junction (TJ) proteins are controlled by the circadian clock; their expression increases during the day and decreases during the night [24]. A diet of large amounts of high-temperature fried foods, associated with obesity, will increase intake of ACR in obese populations, compared to that of the non-obese population. However, it is unclear whether ACR treatment results in chronotoxicity in the livers of HFD-fed mice.

Therefore, the current study was aimed at (a) investigating the survival rates of HFD-fed mice after ACR treatment at ZT0 or ZT12; (b) examining the mitochondrial function in the livers of HFD-fed mice after ACR treatment at ZT0 or ZT12; (c) exploring CYP2E1 expression in the livers of HFD-fed mice after ACR treatment; and (d) investigating the effects of ACR treatment on the gut barrier integrity in HFD-fed mice, and noting any associated liver inflammatory responses. The data from these experiments will assist with revealing the underlying mechanism whereby the diet influences ACR chronotoxicity.

## 2. Results

### 2.1. The Effects of the HFD on the Survival Rate in ACR-Treated Mice at Treated at ZT0 or ZT12

**Figure 1:**
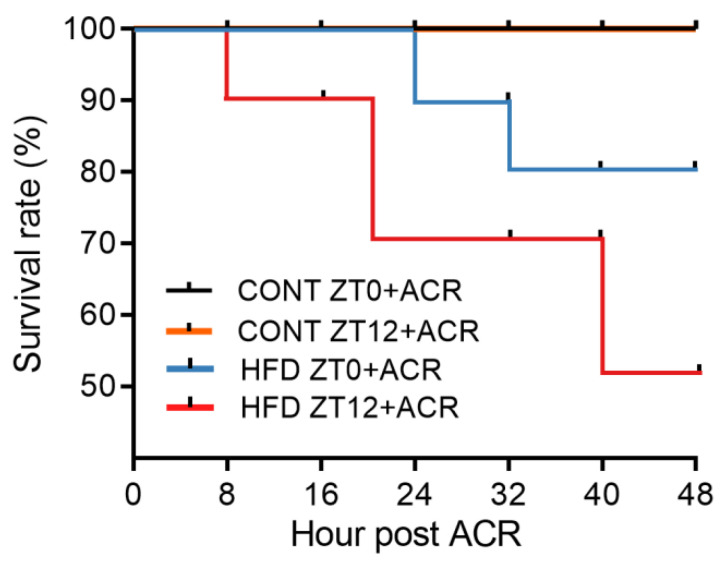
The effects of a HFD on the survival rate in ACR-treated mice at ZT0 and ZT12. A total of 40 mice were randomly divided into 4 groups. The control mice and the HFD-fed mice were treated with 100 mg/kg ACR by means of intragastric administration at ZT0 or ZT12. The survival rate is shown for the four groups of mice after treatment with 100 mg/kg ACR at ZT0 and ZT12 for 48 h (*n* = 10/group).

Previous studies showed that the oral lethal dose (LD50) of ACR for mice was 107–170 mg/kg [25]. To investigate the effects of a HFD on ACR-induced mortality at different time points, the control mice and HFD-fed mice received orally administered doses of 100 mg/kg ACR at ZT0 or at ZT12. The survival rate of these mice was monitored every 8 h for 48 h. As shown in Figure 1, the survival rate of the control group mice was maintained at 100% for 48 h after treating with 100 mg/kg ACR at both ZT0 and ZT12. There was no toxicity in the standard diet that was fed to mice at either ZT0 or ZT12. However, the HFD led to mouse death at 24 h and 16 h after ACR administration at ZT0 and ZT12, respectively. The mortality rate for HFD-fed mice treated at ZT12 was much higher than that of the mice treated as ZT0. After the administration of 100 mg/kg ACR, more than 80% of the mice (HFD ZT0 + ACR) remained alive at 48 h, whereas the survival rate of HFD ZT12 + ACR mice decreased to 50%, suggesting that chronotoxicity existed due to the ACR treatment received by the HFD-fed mice.

### 2.2. The Differential Effects of ACR Administration on Liver Damage at ZT0 and ZT12 in HFD-Fed Mice

**Figure 2:**
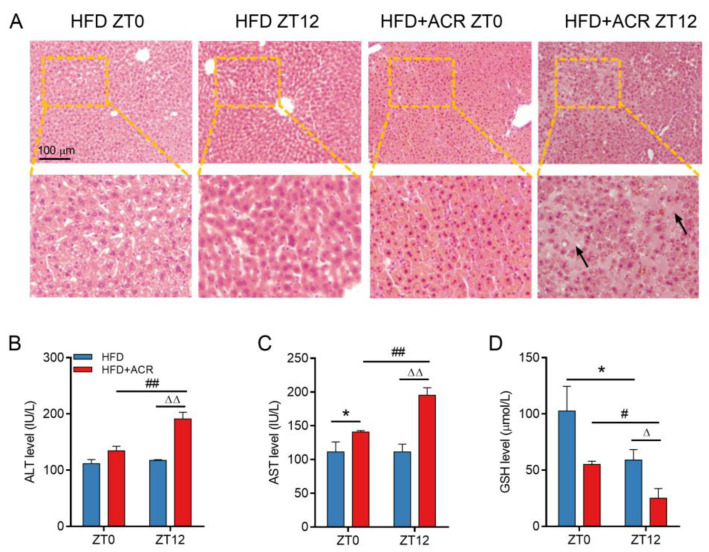
The differential effects of ACR administration on liver damage at ZT0 and ZT12 in HFD-fed mice. A total of 40 mice received a HFD diet to induce an obese model and were then randomly divided into 4 groups (*n* = 10/group). Mice were treated with or without ACR for 7 days at ZT0 or at ZT12. (**A**) Representative H&E staining of the liver and the black arrows indicated the liver fibrosis in HFD + ACR ZT12 group. (**B**,**C**) The serum ALT and AST activity, respectively. (**D**) The serum GSH content. The data are presented as the mean ± SEM, *n* ≥ 6 mice/group. * *p* < 0.05, versus the HFD group at ZT0. ^#^ *p* < 0.05, ^##^ *p* < 0.01, versus the HFD + ACR group at ZT0. ^∆^ *p* < 0.05, ^∆∆^ *p* < 0.01, versus the HFD group at ZT12.

To further investigate the effects of the HFD on ACR-induced liver damage at different time points, the HFD-fed mice received orally administered dose of 25 mg/kg ACR at ZT0 or at ZT12 for 7 days. Histological analysis of liver sections by H&E staining revealed ACR treatment-induced liver fibrosis among HFD-fed mice treated at ZT12, but none inthose treated at ZT0. (Figure 2A). Consistent with these observations, the levels of serum ALT and AST were significantly elevated in HFD-fed mice, and ACR administration at ZT12 further increased the ALT and AST levels compared with the levels in those treated at ZT0 (Figure 2B,C). However, as shown in Figure S1A,B, there was no significant change in serum AST and AST levels between those treated at ZT0 and ZT12 in the standard diet-fed mice. Although ACR significantly increased the ALT and AST levels, there were no significant differences between treatment-time groups, which indicated that there was no chronotoxicity caused by ACR in the livers of mice fed a standard diet. Therefore, in the following study, we focused on investigating the chronotoxic effects of ACR in HFD-fed mice. It was also revealed that the serum GSH levels among HFD-fed mice were much lower for those treated at ZT12 than those treated at ZT0 (Figure 2D). These data indicated that increased hepatic toxicity occurred in a time-dependent manner after ACR treatment in HFD-fed mice.

### 2.3. The Differential Effects of ACR Administration on Liver Mitochondrial Function at ZT0 and ZT12 in HFD-Fed Mice

**Figure 3:**
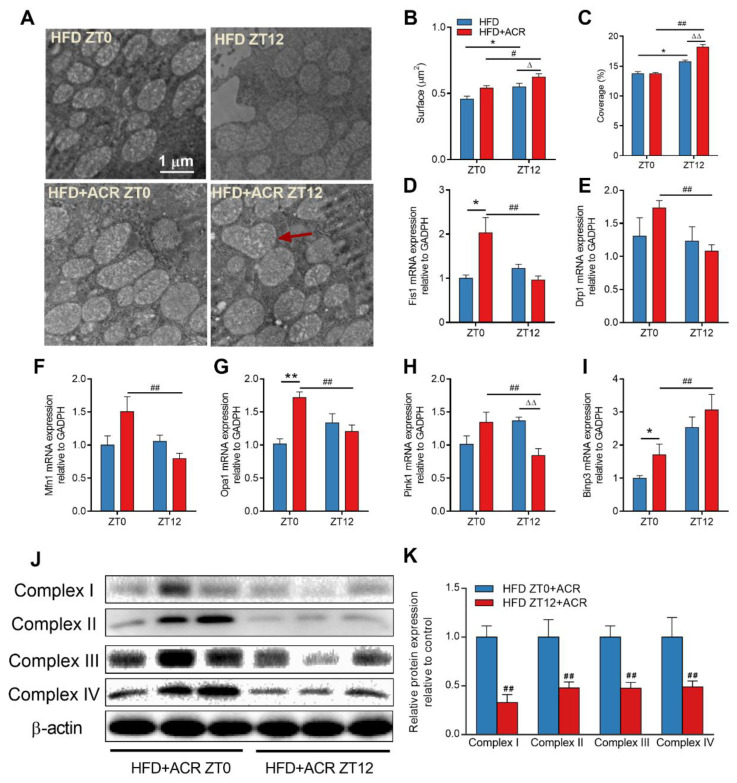
The differential effects of ACR administration on liver mitochondrial function at ZT0 and ZT12 in HFD-fed mice. (**A**) Representative EM images of liver sections from HFD-fed mice with or without ACR treatment at ZT0 and ZT12 (25,000×) and red arrow indicated the swollen phenotype and irregular shape mitochondrial. (**B**,**C**) Mitochondrial surface and coverage, respectively, were calculated from EM images. The mRNA levels of mitochondrial fission, fusion, and mitophagy genes (**D**) Fis1, (**E**) Drp1, (**F**) Mfn1, (**G**) Pink1, (**H**) Opa1, and (**I**) Binp3 were measured by RT-qPCR with or without ACR treatment at ZT0 and ZT12; GAPDH was used as the loading control. (**J**) Representative Western blots of complexes I, II, III, and IV; α-tubulin was used as a loading control. (**K**) The densitometric analyses. The data are presented as the mean ± SEM, *n* ≥ 6 mice/group. * *p* < 0.05, ** *p* < 0.01, versus the HFD group at ZT0. ^#^ *p* < 0.05, ^##^ *p* < 0.01, versus the HFD + ACR group at ZT0. ^∆^ *p* < 0.05, ^∆∆^ *p* < 0.01, versus the HFD group at ZT12.

Electron microscopy was employed to study the ACR-triggered mitochondrial function at the cellular and organelle levels in mice treated at ZT0 and ZT12. As shown in Figure 3A–C, ACR treatment at ZT12 in HFD-fed mice led to a swollen phenotype and irregular mitochondrial shape, as the red arrow indicates. The average surface area and overall coverage (percentage) of mitochondria in the liver were increased in the HFD + ACR ZT12 group. In addition, the mitochondria adapted to different metabolic states by fusion, fission, and mitophagy [26]. To further investigate whether the ACR administration at different time points influenced the mitochondrial dynamics in the livers of HFD-fed mice, the mRNA levels of mitochondrial fission [fission 1 (Fis1) and dynamin-1-like protein (Drp1)], fusion [mitofusin 1 (Mfn1) and optic atrophy 1 (Opa1)], and mitophagy [PTEN-induced putative kinase 1 (Pink1) and BCL2/adenovirus E1B 19 kDa interacting protein 3 (Binp3)] were tested in both groups, ZT0 and ZT12. In Figure 3D–H, compared to the group with ACR treatment at ZT0, the Fis1, Drp1, Mfn1, Opa1, and Pink1 mRNA expression was significantly lower than in the group with ACR treatment at ZT12. In contrast, Figure 3I indicated that Binp3 expression increased with ACR treatment at ZT12. Moreover, the protein levels of mitochondrial respiratory chain complexes I, II, III, and IV in the liver were downregulated in those with ACR treatment at ZT12 among the HFD-fed mice (Figure 3J,K). Above all, the results indicated that, compared to treatment at ZT0, ACR treatment at ZT12 triggers more severe mitochondrial dysfunction in the livers of HFD-fed mice.

### 2.4. The Differential Effects of ACR Administration on CYP2E1 Expression at ZT0 and ZT12 in HFD-Fed Mice

Except for the metabolic pathway involving GSH, ACR was metabolized to GLY, a genotoxic reactive substance that attacks biomacromolecules and is metabolized by the CYP2E1 enzyme in the mouse liver [27]. The mRNA expression of CYP2E1 in the liver of standard diet-fed mice with or without ACR treatment at ZT0 or ZT12 was examined (Figure S2). The current results showed that although ACR treatment enhanced the CYP2E1 mRNA levels for both groups, ZT0 and ZT12, there was no difference between these two groups.

**Figure 4:**
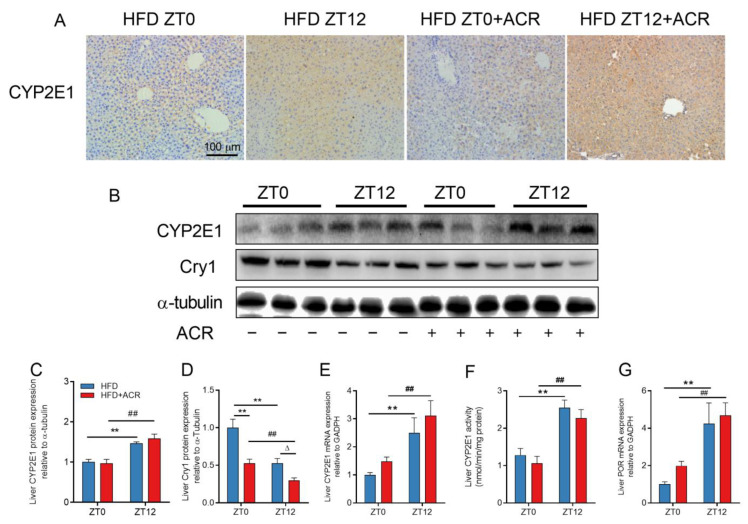
The differential effects of ACR administration on CYP2E1 expression at ZT0 and ZT12 in HFD-fed mice. (**A**) Representative IHC image of CYP2E1 in the mouse liver and the positive cells were stained tan. (**B**) Representative Western blots of CYP2E1 and Cry1 in the mouse liver; α-tubulin was used as a loading control. (**C**,**D**) The densitometric analyses. (**E**) The mRNA level of liver CYP2E1 (**F**) The liver CYP2E1 activity. (**G**) The mRNA level of POR in the mouse liver; GAPDH was used as the loading control. The data are presented as the mean ± SEM, *n* ≥ 6 mice/group. ** *p* < 0.01, versus the HFD group at ZT0. ^##^ *p* < 0.01, versus the HFD + ACR group at ZT0. ^∆^ *p* < 0.05, versus the HFD group at ZT12.

Immunohistochemical staining of CYP2E1 was much deeper in the HFD + ACR ZT12 group, which showed that the CYP2E1 protein expression in the liver at ZT12 was significantly increased, compared to ZT0-ACR-treated, HFD-fed mice (Figure 4A). The western blots of CYP2E1 and liver CYP2E1 activity demonstrated the same tendency (Figure 4B,C,F). However, there was no significant difference in CYP2E1 expression in the gut between the groups treated at ZT0 and ZT12, which was consistent with the mRNA expression (Figure S3A–C). Moreover, nicotinamide adenine dinucleotide phosphate (NADPH)-cytochrome POR, a CYP2E1 rate-limiting enzyme, was also detected in the liver and gut. In ACR-treated mice, mRNA expression of POR among those treated at ZT12 was significantly higher than among those treated at ZT0 (Figure 4E,F). However, there was no significant differences in the mRNA levels of CYP2E1 and POR in the gut (Figure S3D,E). Furthermore, it was demonstrated that the transcription of CYP2E1 was suppressed by circadian protein Cry1, which resulted in a 24-h rhythm of CYP2E1 mRNA expression [28]. The present results showed that the protein expression of Cry1 in the liver significantly decreased among mice treated at ZT12 compared to those treated at ZT0 (Figure 4B,D). ACR treatment further decreased the expression of Cry1 in both groups, but the protein expression was lower for the group treated at ZT12 than for the group treated at ZT0.

**Figure 5:**
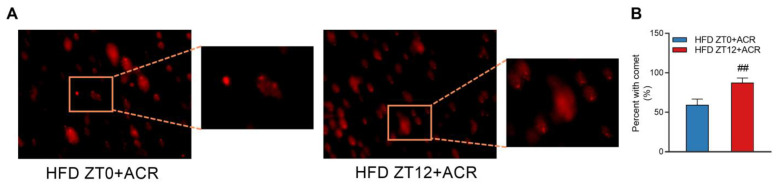
The differential effects of ACR administration on DNA damage in the liver at ZT0 and ZT12 in HFD-fed mice. (**A**) Images of DNA damage as revealed by the comet assay (800×). (**B**) Percent with comet. The data are presented as the mean ± SEM, *n* ≥ 6 mice/group. ^##^ *p* < 0.01, versus the HFD + ACR group at ZT0.

To further investigate the differential effects of ACR treatment on genotoxicity in HFD-fed mice at different time points, the comet assay was conducted to determine the extent of the DNA damage. The results revealed that over 80% of the DNA showed a comet tail in the HFD ZT12 + ACR group, and less than 60% of the DNA had a comet tail in the HFD ZT0 + ACR group (Figure 5A,B). In total, these data revealed that ACR treatment significantly increased the hepatic expression of CYP2E1 and further DNA damage was induced for those treated at ZT12.

### 2.5. The Differential Effects of ACR Administration on the Gut Barrier Integrity and Inflammatory Responses at ZT0 and ZT12 in HFD-Fed Mice

**Figure 6:**
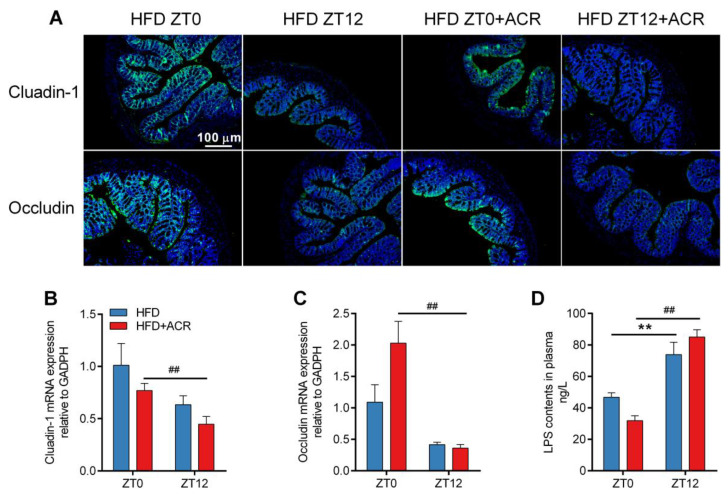
The differential effects of ACR administration on the gut barrier integrity at ZT0 and ZT12 in HFD-fed mice. (**A**) Immunofluorescence staining images of Claudin-1 and Occludin isolated from the mouse gut. The blue fluorescence was DAPI staining and the green fluorescence was fluorescent secondary antibody of Claudin-1 and Occludin. (**B**,**C**) The mRNA levels of Claudin-1 and Occludin, respectively; GAPDH was used as the loading control. (**D**) The LPS content in the serum. The data are presented as the mean ± SEM, *n* ≥ 6 mice/group. ** *p* < 0.01, versus the HFD group at ZT0. ^##^ *p* < 0.01, versus the HFD + ACR group at ZT0.

The gut barrier integrity is controlled by multiple tight junction (TJ) proteins including Claudin-1 and Occludin [29]. It has been revealed that the expression in the gut of Claudin-1 and Occludin proteins and mRNA among mice treated at ZT12 was significantly lower than those treated at ZT0 in ACR-treated, HFD-fed mice (Figure 6A–C). As shown in Figure 6D, the LPS content for those treated at ZT12 was much higher than for those treated at ZT0. However, there were no significant differences in the amounts of serum LPS in standard-fed mice with or without ACR treatment at ZT0 and at Z12 (Figure S4). 

**Figure 7:**
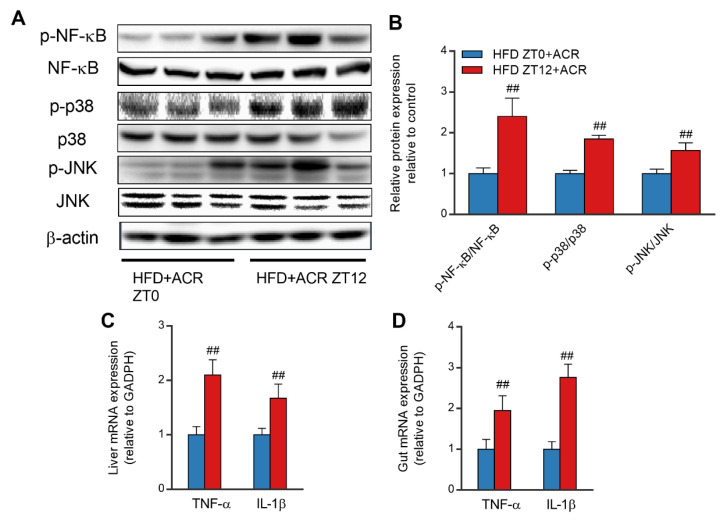
The differential effects of ACR administration on inflammatory responses at ZT0 and ZT12 in HFD-fed mice. (**A**) Western blots with loading controls of p-NF-κB and NF-κB, p-p38 and p38, and p-JNK and JNK. (**B**) The densitometric analyses of p-NF-κB/NF-κB, p-ERK/ERK, p-P38/P38, and p-JNK/JNK. (**C**) The mRNA levels of TNF-α and IL-1β in the livers of ACR-treated, HFD-fed mice at ZT0 and ZT12; GADPH was used as the loading control. (**D**) The mRNA levels of TNF-α and IL-1β in the gut of ACR-treated, HFD-fed mice at ZT0 and ZT12; GADPH was used as the loading control. The data are presented as the mean ± SEM, *n* ≥ 6 mice/group. ^##^ *p* < 0.01, versus the HFD + ACR group at ZT0.

To further investigate the day and night differences in the inflammatory responses of the livers of ACR-treated, HFD-fed mice, the phosphorylation of NF-κB was determined for the ZT0 and ZT12-treatment groups. The phosphorylation of NF-κB in ACR-treated, HFD-fed mice treated at ZT12 was much more activated compared to those treated at ZT0 (Figure 7A,B). Similarly, the phosphorylation of c-Jun N-terminal kinase (JNK) and p38 were also significantly higher for the ZT12-treatment group. Furthermore, the mRNA levels of TNF-α and IL-1β in the gut and liver were significantly higher for the ZT12-treatment group (Figure 7C,D). In summary, the HFD significantly inhibited the expression of gut TJ proteins in mice, which was increased by ACR treatment at ZT12, and further induced LPS leakage and inflammatory responses in both the gut and liver.

## 3. Discussion

Here, we found that the higher dose (100 mg/kg) of ACR induced a decrease in the mouse survival rate in HFD-fed mice, but not in standard diet-fed mice, with an increased effect observed among those treated at ZT12. ACR administration at ZT12 was found to trigger more severe liver damage including mitochondrial dysfunction as compared to ZT0 administration in HFD-fed mice. ACR also provoked more DNA damage at ZT12 compared to ZT0, which could be partly explained by the HFD leading to higher hepatic expression of the CYP2E1 enzyme at ZT12. Moreover, the expression in the gut of Claudin-1, the TJ protein, was lower at ZT12 in mice that received the HFD with acrylamide treatment, which led to gut leakage and inflammatory responses in both the gut and liver.

The recent study revealed that the typical human daily intake of acrylamide was from 0.02–1.53 μg/kg [30,31]. For our animal experiments, we referred to several previous studies to select an appropriate dosage. For example, Park and Kim et al. used 10 mg/kg/day and 50 mg/kg/day ACR to explore whether ACR impaired hippocampal neurogenesis [32]. In addition, a recent study revealed that 25 mg/kg/day and 50 mg/kg/day ACR could induced mice liver oxidative stress [33]. The current research also used 25 mg/kg/day ACR to treat HFD mice at ZT0 and ZT12, and found that HFD mice suffered more damage if treated at ZT12 than if treated at ZT0. Although the dose of ACR used in our experiment is much higher than the typical human daily intake of ACR, it still could assist with revealing the underlying mechanism whereby the diet influences ACR chronotoxicity. 

Previous research found that long-term acrylamide treatment induced liver damage and circadian oscillation disorders via suppression of circadian gene expression [1]. Moreover, we also found that ACR caused microglial mitochondrial dysfunction by reducing membrane potential, inhibiting mitochondrial complex expression, and decreasing mitochondrial respiration [3]. Mitochondria are the primary energy organelle that generates adenosine 5′-triphosphate (ATP) via oxidative phosphorylation [34]. A HFD has been reported to induce liver mitochondrial dysfunction, including downregulating mitochondrial biogenesis, mitochondrial respiration, ATP synthesis, and mitochondrial dynamics [26,35,36]. The mitochondrial dynamics, i.e., fusion and fission, are mediated by circadian rhythm genes [26]. We found that the average surface area and the overall coverage (percentage) of mitochondria in the liver at ZT12 were significantly higher than at ZT0 in HFD-fed mice. Moreover, ACR administration at ZT12 triggered mitochondrial swelling, structural damage, and even higher surface area and overall coverage than did administration at ZT0 in HFD-fed mice (Figure 3A–C). These results indicated that ACR confers chronotoxicity to mitochondrial function in HFD-fed mice.

As previously mentioned, ACR is mainly metabolized by two pathways, i.e., the GSH conjunction pathway and the CYP2E1 enzyme metabolism pathway. It has been previously demonstrated that a HFD leads to oxidative stress in the liver by generating excessive ROS and reducing GSH levels in obese mice [15,37]. The GSH level, however, is also regulated by the circadian clock. Rodent studies demonstrated that GSH expression has an acrophase (time of peak expression) during daylight hours [38]. As mentioned above, ACR treatment decreased GSH levels. Consistent with our current research, we found that ACR-treated, HFD-fed mice exhibited significantly reduced levels of GSH at ZT12 as compared to ZT0 (Figure 2D).

The CYP2E1 enzyme in the liver mediates the metabolism of ACR into GLY, which is more toxic than acrylamide because it readily attacks DNA structure and consumes GSH at a rate that is 1.5-fold faster than that of ACR [39]. In our previous study, we found that a HFD induced glycolipid metabolism disorders and further triggered circadian dysfunction by introducing imbalance to circadian clock gene expression [17]. In addition, CYP2E1 played a key role in the hepatic toxicity of xenobiotic, including toxins and drugs, and high CYP2E1 expression was usually detrimental for liver xenobiotic metabolism [40]. CYP2E1 is able to activate numerous chemicals, is required for the hepatotoxicity of toxins, and is also thought to act on hepatic injury through produced ROS [41]. It has been found that the expression of CYP2E1 is transcriptionally regulated by circadian gene Cry1, which indicates that the metabolism of drugs and xenobiotic by CYP2E1 might also be regulated by circadian genes [28]. Moreover, it has been reported that the hepatic expression of CYP2E1 in high-fat diet-induced obese rats was upregulated [20,41]. 

A recent finding demonstrated that there were no significant differences in the protein levels of CYP2E1 between ZT0 and ZT12 in normally fed mice [42]. However, in the current work, we found that the HFD significantly elevated the hepatic protein expression of CYP2E1 in mice at ZT12 (Figure 4A–C), which was accompanied by the increased expression of POR, a rate-limiting enzyme of CYP2E1 expression. The upregulated expression of CYP2E1 might increase the generation of GLY, which was confirmed by our finding in the comet experiment that the DNA damage in the liver was more severe at ZT12 than at ZT0 in HFD-fed mice (Figure 5). Interestingly, we also found that the protein level of circadian gene Cry1 was lower in ACR-treated mouse livers at ZT12 as compared to ZT0 (Figure 4B,D). Although the dosage of ACR used in this study represents extreme acute hepatotoxicity and is well above the theoretical daily intake, it still provides a proof of concept that these obese populations may be at differential risk from exposure to hepatotoxicants such as ACR. In addition, even though the mice were nocturnal animals and the circadian clock may be contrary to human beings, these results indicated that a HFD disturbed the circadian rhythm metabolism and upregulated CYP2E1 expression in mice liver at night, which consequently increased the hepatotoxicity of ACR. This partly explains how ACR causes chronotoxicity in the livers of HFD-fed mice.

Previous research indicated that the cross-talk between the gut and liver could be partly explained by the liver receiving most of its blood supply from the gut through the portal vein, and gut-derived toxic factors, including bacteria and LPS [43,44]. The accumulation of LPS further activates the immune cells and upregulates the expression of inflammatory mediators including TNF-α and IL-1β via stimulating the NF-κB pathway, which leads to liver damage [45]. It has been found that a HFD elevated lipogenesis and lipid accumulation in the jejunum, which consequently triggered oxidative stress and gut barrier damage [46,47]. A HFD has also been found to suppress TJ protein expression, which further increases the permeability of the gut and allows increased transport of LPS from the gut to serum [1]. Previous research found that acrylamide treatment also significantly inhibited the expression of TJ protein [2]. In the present study, however, we demonstrated that ACR downregulated the expression of Claudin-1 and Occludin at ZT12 than that at ZT0 in HFD-fed mice (Figure 6A–C). Consistently, the levels of LPS in serum were significantly higher at ZT12 than at ZT0 in the ACR-treated HFD-fed mouse group (Figure 6D). The treatment at ZT12 also dramatically stimulated the phosphorylation of NF-κB as compared to treatment at ZT0 (Figure 7A,B). These results indicated that ACR treatment at ZT12 increased inflammatory responses via degradation of the gut barrier integrity in HFD mice.

## 4. Materials and Methods

### 4.1. Animals and Treatments

Male C57BL/6J mice (3-month-old) were purchased from Xi’an, Jiaotong University (Xi’an, China). Acrylamide (purity > 99%) was purchased from Sigma, Ltd. (St. Louis, MO, USA). Mice were housed in an animal facility under standard conditions (12/12 light-dark cycle, humidity at 5 0 ± 15%, temperature at 2 2 ± 2 °C). The mice were randomly divided into 12 groups (*n* = 10/group).

Experiment 1: The mice were divided into four groups of mice called the CONT ZT0 + ACR group, CONT ZT12 + ACR group, HFD ZT0 + ACR group, and HFD ZT12 + ACR group, and they were fed with the control diet or the HFD. The CONT group mice were fed with a standard diet (AIN-93M), and the HFD group received a high-fat diet (45% kcal from fat, TP230100, purchased from TROPHIC Animal Feed High-tech Co., Ltd. Nantong, China) for 10 weeks. The average body weights of the CONT group mice and HFD group mice were 25.8 g and 34.2 g, respectively. A higher oral dose of ACR (100 mg/kg, dissolved in 100 μL saline) was administered to the CONT group mice and the HFD-fed mice at zeitgeber-time (ZT) 0 (night-day transition) or ZT12 (day-night transition), according to their group designation; 8:00 a.m. was recognized as ZT0 and 8:00 p.m. was recognized as ZT12. The survival rate was then recorded at 48 h.

Experiment 2: The mice were divided into 8 groups called the CONT ZT0 group, CONT ZT12 group, CONT ZT0 + ACR group, CONT ZT12 + ACR group, HFD ZT0 group, HFD ZT12 group, HFD ZT0 + ACR group, and HFD ZT12 + ACR group. Saline or a lower dose of ACR (25 mg/kg, dissolved in 100 μL saline) was orally administered to the 8 groups of mice at ZT0 or at ZT12 for 7 days, according to their group designation. Then, the mice were euthanized 3 h later on day 7 after the final intragastric administration. All animal experiments complied with the ARRIVE guidelines and were carried out in accordance with the U.K. Animals (Scientific Procedures) Act, 1986 and associated guidelines, EU Directive 2010/63/EU. Animal experiments were performed in accordance with the guidelines of the Shandong Agricultural University Animal Care and Use Committee (permit No. SDAU 18-096; 6 July 2018). All of the surgeries were performed under anesthesia, and all efforts were made to minimize suffering.

### 4.2. RNA Preparation and RT-qPCR

The RNA preparation and RT-qPCR were carried out as previously described [48]. Briefly, total RNA was isolated from the liver and gut using a TRIzol Kit (Genshare Biological TRIzol Kit, Xian, and China). RNA samples were obtained from the HFD-fed mice with or without ACR treatment at ZT0 or at ZT12. RNA (1 mg) was reverse transcribed into cDNA using the Primescript RT Master Mix reverse transcription kit (Takara Primescript RT master Mix, Dalian, and China). cDNA was stored at −80 °C for further analysis by microarray and real-time quantitative PCR (RT-qPCR). Then, the mRNA levels were quantified by RT-qPCR using the CFX96TM real-time system (Bio-Rad, Hercules, CA, USA). Gene-specific mouse primers were used as described in Table 1. Ct values were normalized to GAPDH, and the relative gene expression was calculated with the 2^−△△Ct^ method (*n* = 6/group/time point).

### 4.3. Western Blots

The Western blots were performed as described in the previous study [1,49]. The liver and gut tissue homogenates were solubilized in sodium dodecyl sulfate (SDS) sample buffer. Samples were separated by SDS-polyacrylamide gel electrophoresis (PAGE) and transferred onto polyvinylidene difluoride (PVDF) membranes. Appropriate primary antibodies were used for incubating overnight. Antibodies against mitochondrial respiratory chain complex I (NADPH-diaphorase) (SC-20493), II (complexII) (sc65239), complex III (cytochrome reductase) (SC-69064), β-actin (SC-47778) were purchased from Santa Cruz Biotechnology (Santa Cruz, CA, USA). Antibodies against CYP2E1 (ab28146), Cry 1 (ab54649), α-tubulin (ab7291), p-NF-kB (ab76302), NF-kB (ab76311), p38 (ab182453), p-p38 (ab178867), JNK (ab199380), p-JNK (ab307802) were purchased from Abcam (Abcam, Cambridge, MA, USA). The dilution concentration of the primary antibody is 1:1000. The immunoreactive bands were visualized with an enhanced chemiluminescence (ECL) reagent after incubating appropriate secondary antibodies (dilution concentration is 1:5000). Quantification of Western blot results using band densitometry analysis was performed with Quantity One software 4.6.2.

### 4.4. Serum Analysis

The serum total alanine aminotransferase (ALT, C009-2), aspartate aminotransferase (AST, C010-2), and reduced glutathione (GSH, A006-2) levels were measured using enzymatic assay kits (Nanjing Jiancheng Bioengineering Institute, Nanjing, China). The LPS in the serum, liver, and gut was tested using a commercial ELISA kit (Mouse LPS kit, Xinle Biology Technology, Shanghai, China).

### 4.5. Measurement of Hepatic CYP2E1 Activity

Liver tissues from different groups were homogenized in 50 mM Tris with 1.15 (*w*/*v*) KCl. Then adjusted PH value to 7.4. The homogenized samples were centrifuged for 20 min at 4 °C with 10,000 *g*/min. The protein concentration of the final supernatant was determined by the BCA protein assay kit and the final protein concentrion was 1 μg/μL. CYP2E1 activity was determined by measuring the change ratio of 4-nitrophenol to 4-nitrocatechol and expressed as nmol/min/mg protein, and absorbance at 510 nm was measured by microplate reader (Bio-Rad Laboratories, Ltd., Shanghai, China)

### 4.6. Hematoxylin and Eosin (H&E), Immunohistochemical (IHC), and Immunofluorescence Staining

Gut and liver tissues were embedded in paraffin for staining with hematoxylin and eosin. The procedures for immunohistochemical and immunofluorescence staining were performed as described in a previous study [50]. Briefly, the fixed liver and gut sections were exposed to the primary antibodies at 4 °C overnight. After incubation, the sections were washed three times with PBS and incubated with the appropriate secondary antibody or fluorescent secondary antibody. The images were obtained by inverted fluorescence microscope at 400× (Olympus, Tokyo, Japan).

### 4.7. Structural Analysis of the Liver Samples by Transmission Electron Microscopy (TEM)

Liver samples were cut into small pieces (approximately 1 mm^3^) and immediately placed in a cold fixative solution composed of 2.5% glutaraldehyde in PBS (0.1 mol/L, pH 7.2) at 4 °C. The sample preparation procedures were performed according to previously described methods [5]. Briefly, after fixing the samples in 1% osmium tetroxide (in 0.2 mol/L PBS, pH 7.2) and subjecting them to a dehydration series, the samples were embedded overnight in a mixture of LR-White resin (London Resin Company, Reading, UK) and alcohol (1:1, *v*/*v*). Then, pure LR-White resin was used for embedding, and the samples were incubated at 60 °C for 48 h. All of the fixed sections were examined under a JEM-1230 transmission electron microscope (JEOL, Tokyo, Japan) at 80 kV, and all of the images were recorded with a BioScan Camera Model 792 (Gatan, Pleasanton, CA, USA).

### 4.8. Comet Assay

The comet assay was performed according to the instructions supplied with the assay kits (Nanjing Jiancheng Bioengineering Institute, Nanjing, China). Briefly, the liver tissue was prepared so that a cell suspension was formed. The cell suspension and agarose gel were mixed on glass slides, and the cells were then treated with pre-cooled lysis buffer for 2 h. The glass slides were placed in a horizontal electrophoresis tank and electrophoresed for 30 min at 25 V. Then, the cells were stained with propidium iodide (PI). The migrated DNA (the comet tails) was observed with an inverted fluorescence microscope at 800× (Olympus, Tokyo, Japan).

### 4.9. Data Analysis

The data are reported as the mean ± standard error of the mean (SEM) from at least three independent, repeated experiments. Significant differences between the HFD ZT0 + ACR and HFD ZT12 + ACR groups were determined by one-way analysis of variance (ANOVA) using GraphPad 6.0 software. Significant differences between mean values with treatment (saline/ACR) and two different time points (ZT0 and ZT12) were analyzed by two-way ANOVA, followed by Tukey’s test (GraphPad Prism 6.0). Mean values were statistically significant if *p* < 0.05.

## 5. Conclusions

In conclusion, our study indicated that ACR treatment resulted in chronotoxicity in HFD-fed mice, which could be possibly explained by an increase in the expression of CYP2E1 due to the HFD, which led to more severe toxicity and gut leakage that further stimulated liver inflammatory responses during the night. These data revealed that an unhealthy diet could also impair CYP2E1 metabolism of toxins in food and drugs.

## Figures and Tables

**Table 1:** Primer Sequences Used for qRT-PCR analysis.

	Forward Primer	Reverse Primer
Fis1	AGGCTCTAAAGTATGTGCGAGG	GGCCTTATCAATCAGGCGTTC
Drp1	CGTGACAAATGAAATGGTGC	CATTAGCCCACAGGCATCAG
Pink1	CTGAGATGCCTGAGTCGGTG	CTGAGATGCCTGAGTCGGTG
Binp3	CTCCCAGACACCACAAGATAC	CTTCCTCAGACAGAGTGCTG
Mfn1	CCTACTGCTCCTTCTAACCCA	AGGGACGCCAATCCTGTGA
Opa1	CTGAGGCCCTTCTCTTGTTAGG	CTGACACCTTCCTGTAATGCTTG
CYP2E1	CCTGGTGGAGGAGCTCAAAA	TGTTGAAGAGAATATCCGCAATGA
POR	AGGCACATCCTAGCCATTCTCCAA	ACTTCGCTTCATACTCCACAGCCA
ZO-1	TGGGAACAGCACACAGTGAC	GCTGGCCCTCCTTTTAACAC
Occludin	ACCCGAAGAAAGATGGATCG	CATAGTCAGATGGGGGTGGA
Claudin-1	CGGGCAGATACAGTGCAAAG	ACTTCATGCCAATGGTGGAC
Tnf-α	CCCTCACACTCAGATCATCTTCT	GCTACGACGTGGGCTACAG
IL-1β	TGACGGACCCCAAAAGATGA	TCTCCACAGCCACAATGAGT
GADPH	TGGAGAAACCTGCCAAGTATGA	TGGAAGAATGGGAGTTGCTGT

## Data Availability

Data are presented within the manuscript. Additional raw data are available on request from the corresponding author.

## Supplementary Materials

The following supporting information can be downloaded at: https://www.mdpi.com/article/10.3390/molecules28135132/s1. Figure S1: The differential effects of ACR administration on serum ALS and AST levels at ZT0 and ZT12 in standard diet-feeding group mice; Figure S2: The differential effects of ACR administration on liver CYP2E1 expressions at ZT0 and ZT12 in standard diet-feeding group mice; Figure S3: The differential effects of ACR administration on gut CYP2E1 expressions at ZT0 and ZT12 in HFD-feeding mice; Figure S4: The differential effects of ACR administration on serum LPS contents at ZT0 and ZT12 in standard diet-feeding group mice.
