# Supplementary material for: Chronotoxicity of Acrylamide in Mice Fed a High-Fat Diet: The Involvement of Liver CYP2E1 Upregulation and Gut Leakage
Source: Molecules. 2023 Jun 30;28(13):5132. doi: 10.3390/molecules28135132 (PMC10343525; doi:10.3390/molecules28135132)
Supplement: Supplementary file 1 [file molecules-28-05132-s001.zip › molecules-2429870-supplementary.pdf]

Supplementary materials

Supplemental Figure 1

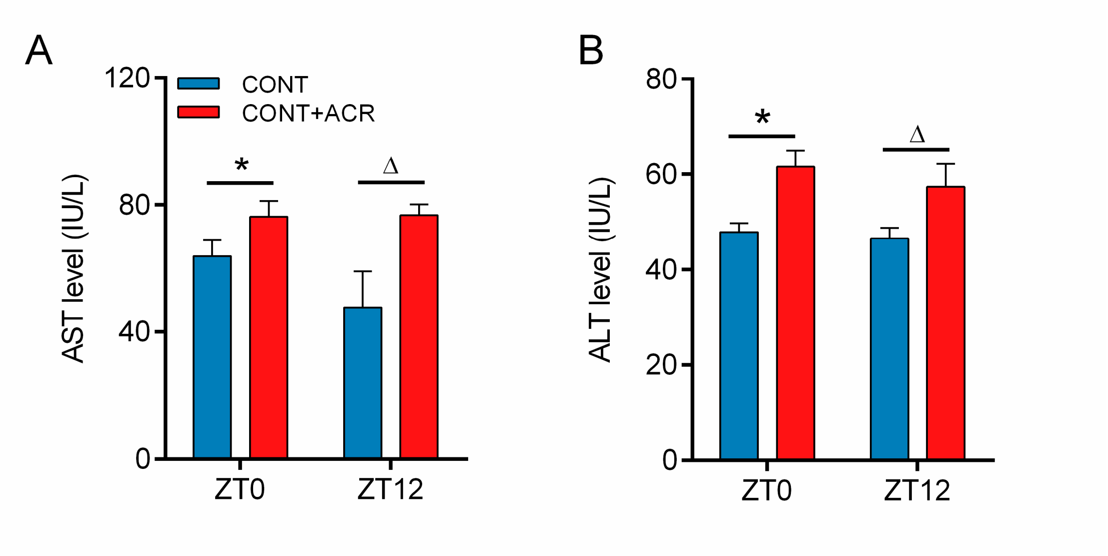

Figure S1. The differential effects of ACR administration on serum ALS and AST levels at ZT0 and ZT12 in standard diet-feeding group mice. The CONT group mice, fed with standard diet, were orally administrated with saline or a lower dose of ACR (25 mg/kg) at ZT0 and ZT12 for 7 days. (A) The serum AST levels; (B) The serum ALT levels; Data presented as mean ±SEM, n≥6 mice/group. *$p<0.05$, versus CONT group at ZT0. Δ$p< 0.05$, versus HFD group at ZT0.

Supplemental Figure 2

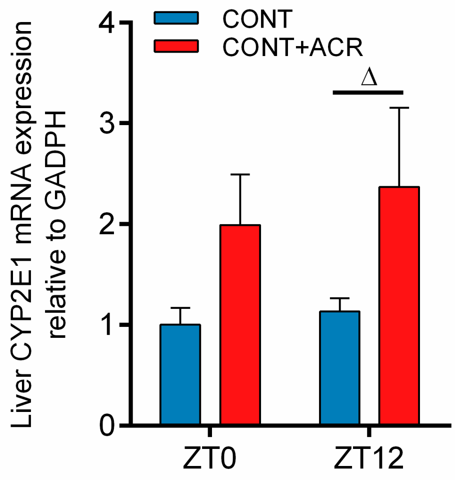

Figure S2. The differential effects of ACR administration on liver CYP2E1 expressions at ZT0 and ZT12 in standard diet-feeding group mice. The CYP2E1 mRNA expressions were determined in CONT group mice liver with or without ACR treatment at ZT0 and ZT12. Data presented as mean ±SEM, n≥6 mice/group. Δ$p < 0.05$, versus HFD group at ZT0.

Supplemental Figure 3

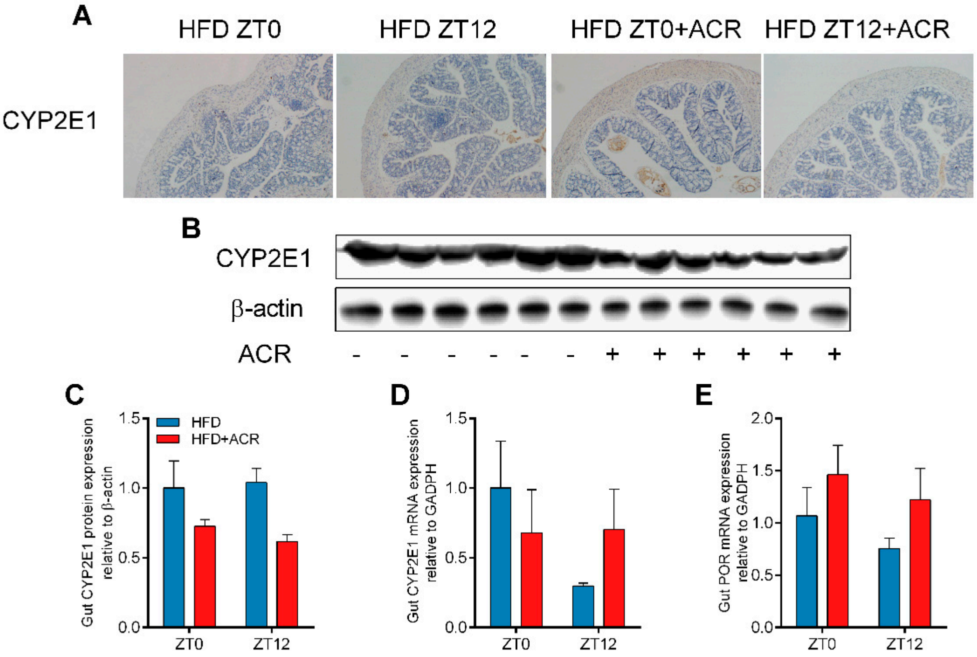

Figure S3. The differential effects of ACR administration on gut CYP2E1 expressions at ZT0 and ZT12 in HFD-feeding mice. (A) Representative IHC image of CYP2E1 in mice gut. (B) Representative western blots of CYP2E1 in mice gut, β-actin was used as a loading control. (C) The densitometric analyses. (D)-(E) The mRNA level CYP2E1 and POR in mice gut. GAPDH was used as the loading control. Data presented as mean ±SEM, n≥6 mice/group.

Supplemental Figure 4

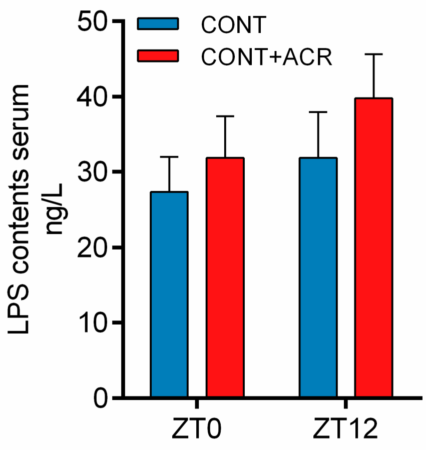

Figure S4. The differential effects of ACR administration on serum LPS contents at ZT0 and ZT12 in standard diet-feeding group mice. The serum LPS contents were determined in CONT group mice liver with or without ACR treatment at ZT0 and ZT12. Data presented as mean ±SEM, n≥6 mice/group.
